# *Cornus × elwinortonii* and *Cornus × rutgersensis* (Cornaceae), new names for two artificially produced hybrids of big-bracted dogwoods

**DOI:** 10.3897/phytokeys.55.9112

**Published:** 2015-08-05

**Authors:** Robert Mattera, Thomas Molnar, Lena Struwe

**Affiliations:** 1 Department of Plant Biology and Pathology, Rutgers University, 59 Dudley Road, New Brunswick, NJ 08901, USA; 2 Graduate Program in Plant Biology, Rutgers University, 59 Dudley Road, New Brunswick, NJ 08901, USA; 3 Department of Ecology, Evolution, & Natural Resources, Rutgers University, 14 College Farm Road, New Brunswick, NJ 08901, USA

**Keywords:** Cornaceae, East Asia, horticulture, hybridization, nomenclature, North America

## Abstract

Big-bracted dogwoods (*Cornus* sp.) are well-known plants in North America and eastern Asia where they occur as wild, generally spring-flowering understory trees. They are also popular ornamental landscape plants, and many economically important cultivars are propagated and sold across North America, Europe, and Asia. Starting in the late 1960s, Elwin Orton of Rutgers University in New Jersey (USA) utilized three geographically disjunct species of dogwoods, *Cornus florida* (eastern North America), *Cornus nuttallii* (western North America), and *Cornus kousa* (East Asia), in an extensive interspecific hybridization program. He was successful in developing the first-ever interspecific F_1_ hybrids of these species, several of which have become staple items in the ornamental nursery trade due to their enhanced ornamental qualities and resistance to diseases. The original F_1_ plants are still alive at Rutgers University. While they have been available for decades in horticultural commerce, the interspecific hybrid crosses were never formally described and their scientific hybrid names were never published. For the *Cornus kousa* × *Cornus florida* hybrids, the name *Cornus* ‘rutgersensis’ has been used on occasion in the horticultural trade, but without proper citation and description. Here, it is formally named Cornus × rutgersensis Mattera, T. Molnar, & Struwe, **hybr. nov.** For the *Cornus kousa* × *Cornus nuttallii* hybrids, no previous name has been used, and it is hereby named Cornus × elwinortonii Mattera, T. Molnar, & Struwe, **hybr. nov.** The need for providing scientific names for commonly used horticultural hybrids is discussed. Holotype material for both hybrid names was collected from the original F_1_ hybrids for full documentation, typification, and description. The comparative intermediate development of leaves, inflorescence structures, and fruit types of the hybrids and their parents is discussed and illustrated. Etymology, phenology, and cultivation aspects of these hybrids and their cultivars including backcrosses to *Cornus kousa* are also presented.

## Introduction

The circumboreal genus *Cornus* L. (Cornaceae, Cornales; APG III 2009) contains about 60 species divided into ten subgenera (Fan and Xiang 2001). Species in this genus express a wide variety of morphologies, from low herbaceous ground covers, such as the boreal-temperate species *Cornus suecica* L., to multi-stemmed shrubs, such as *Cornus sericea* L. It also includes small to large trees, such as *Cornus kousa* Buerger ex Miq. and *Cornus nuttallii* Audubon ex Torr. & A.Gray, the latter of which can grow up to 24 m tall. Some taxonomists have divided the genus up into six genera, but molecular studies have shown that *Cornus* in the current circumscription is monophyletic (Xiang et al. 2006).

Several species of *Cornus* have large, showy petaloid bracts located under tight head-like, multi-flowered inflorescences. These species form the monophyletic big-bracted (BB) clade sensu Xiang et al. 2006, and are mostly spring-flowering trees of North American and East Asian forests. The members of this clade are classified into three different subgenera: *Cynoxylon*, *Discocrania*, and *Syncarpea* (Xiang et al. 2006). The most commonly known big-bracted species in North America are *Cornus florida* L. and *Cornus nuttallii* of subgenus *Cynoxylon* and *Cornus kousa* of subgenus *Syncarpea*. Seed and clonally propagated big-bracted dogwoods are popular ornamental landscape trees in subtropical to temperate regions around the world. Their most conspicuous characteristics are their large, white or red petaloid floral bracts, showy red fruits, and brightly colored fall foliage (Li et al. 2009). *Cornus kousa* can be easily distinguished from the other two species by its round, fleshy multiple fused fruits formed from a whole flower head (as opposed to single, separate drupes from each flower arranged in clusters). It can also be identified by its acute or acuminate floral bracts, whereas the others have bracts that are rounded or retuse (Harrison 2009).

Typical horticultural uses of the big-bracted dogwoods include container, specimen, or shade plantings in suburban landscapes, display gardens, and parks (Gilman and Watson 1993a, b, Mohlenrock 2006). In the eastern and southeastern USA, *Cornus florida* is a common component of native deciduous forests, gardens, and home landscapes. It is among the first trees to bloom with conspicuous flowers in the spring in North America, with a range of cultivars available that express dwarf to vigorous growth habits and white, pink or red floral bracts. *Cornus kousa* is also a common component of ornamental landscapes in the eastern USA. It blooms about a month later than *Cornus florida* (after the leaves have developed), has a more vase-shaped growth habit, and most have white floral bracts, although a few forms with light pink bracts exist (Cappiello and Shadow 2005, Dirr 2009, Rhoades et al. 2011). The use of *Cornus nuttallii* in landscaping is much more limited than the former two species, due to limited winter hardiness in the eastern USA and it is cultivated mostly in the Pacific Northwest (USA), where it is native. Dogwood sales in the USA account for over 11% of the total deciduous flowering tree market, amounting to nearly 31 million USD in 2009 (Fulcher et al. 2012, NASS 2007).

The Rutgers University dogwood breeding program began in 1965 under the direction of horticultural plant breeder Dr. Elwin Orton. The early goals of the program were to develop novel cultivars of *Cornus florida* and *Cornus kousa* with improved aesthetic qualities, including pink and red floral bracts, unique growth habits, and superior disease resistance. Several years after the program started, attention was turned toward developing interspecific hybrids between these two species as well as between *Cornus kousa* and *Cornus nuttallii*, to help reach these goals (Elwin Orton personal communication). Because of differences in flowering times between the species, which can span more than a month, Orton used two approaches to make the hybrid crosses. First, he collected, dried, and stored pollen from earlier flowering plants to apply to the stigmas of those that bloomed later in the field and greenhouse. Second, he manipulated bloom times through the careful use of cold chambers and warm greenhouses to artificially break dormancy and match flowering times of container-grown plants to those in the field (E. Orton personal communication). Orton was ultimately successful in his interspecific hybridization attempts and is credited as being one of the first to create *Cornus florida* × *Cornus kousa* and *Cornus kousa* × *Cornus nuttallii* F_1_ hybrids (Dirr 2009). To date, eleven interspecific cultivars, comprising eight from *Cornus florida* × *Cornus kousa* crosses and three from *Cornus kousa* × *Cornus nuttallii* hybrids, have been named, released, and patented through the Rutgers University dogwood breeding program (Table 1). The two classes of interspecific hybrids display intermediate morphological and phenological characteristics between the parental species (Cappiello and Shadow 2005, Dirr 2009, E. Orton personal communication, Orton 1990a, 1990b, 1990c, 1990d, 1990e, 1991, 2014, Orton and Gant 1993a, 1993b, 2006a, 2006b, 2004, 2007, 2011). Many also show increased vigor (rates of growth) compared to their parent species, as well as improved stress tolerance.

**Table 1.** Parentage of the eleven interspecific hybrids released from the Rutgers University dogwood breeding program. PP refers to plant patent number. OP indicates open pollination. Brackets ([ ]) contain pedigree information of an interspecific hybrid parent.

Scientific name	Cultivar, Patent number, Trademark	Female parent	Male parent
Cornus × elwinortonii	‘KN30-8’, PP 16309, Venus® (Jersey Star® Series)	[*Cornus kousa* ‘Chinensis’ × *Cornus nuttallii* ‘Goldspot’]	*Cornus kousa* ‘Rosea’
Cornus × elwinortonii	‘KN4-43’, PP 16293, Starlight® (Jersey Star® Series)	*Cornus kousa* ‘Simpson No. 1’	*Cornus nuttallii* ‘Goldspot’
Cornus × elwinortonii	‘KN144-2’, PP application number 2014-0283242, Rosy Teacups®	[*Cornus kousa* ‘Chinensis’ × *Cornus nuttallii* ‘Goldspot’] × OP	*Cornus kousa* ‘Rosabella’
Cornus × rutgersensis	‘KF111-1’, PP 22219, Hyperion®	*Cornus kousa* K2 × *Cornus florida* ‘Sweetwater Red’	Unknown
Cornus × rutgersensis	‘KF1-1’, PP 17768, Saturn®	*Cornus kousa* K2	*Cornus florida* D1
Cornus × rutgersensis	Cornus ‘Rutlan’, PP 7732, Ruth Ellen® (Stellar® Series)	*Cornus kousa* K2	*Cornus florida* ‘Meyer White’
Cornus × rutgersensis	Cornus ‘Rutfan’, PP 7206, Stardust® (Stellar® Series)	*Cornus kousa* K2	*Cornus florida* ‘Cherokee Princess’
Cornus × rutgersensis	Cornus ‘Rutcan’, PP 7210, Constellation® (Stellar® Series)	*Cornus kousa* K2	*Cornus florida* ‘Cherokee Princess’
Cornus × rutgersensis	Cornus ‘Rutdan’, PP 7204, Celestial® (Stellar® Series)	*Cornus kousa* K2	*Cornus florida* D1
Cornus × rutgersensis	Cornus ‘Rutban’, PP7205, Aurora® (Stellar® Series)	*Cornus kousa* K2	*Cornus florida* ‘Springtime’
Cornus × rutgersensis	Cornus ‘Rutgan’, PP7207, Stellar Pink® (Stellar® Series)	*Cornus kousa* K2	*Cornus florida* ‘Sweetwater Red’

According to the *International Code of Nomenclature for algae, fungi, and plants* (abbreviated hereafter as ICN), a hybrid between two plant species can be given two types of scientific names to classify them within the taxonomic system of plant biodiversity (McNeill et al. 2012: Art. H1). Either the hybrid is listed with the name of the two parents separated by a multiplication (×) sign, such as in the oak hybrid *Quercus alba* × *Quercus bicolor*, or they may be given a unique name with the species epithet preceded by a multiplication (×) sign, such as Quercus × jackiana for the same hybrid (Haines 2011). For hybrids in horticulture and commerce, the second option is preferred since it provides a simpler name that is easier for horticulturalists and the public to learn, catalogue, use on labels, and remember. It also provides a scientific name that fits into existing databases already in use for commercial plants. For the two flowering dogwood hybrids discussed here, no formal scientific names have been proposed, although ‘Cornus × rutgersiensis’ and ‘Cornus × rutgersensis’ (sometimes without the multiplication sign, ×) have been used in popular and horticultural literature for many years to indicate *Cornus florida* × *Cornus kousa* hybrids (e.g., Gayraud 2013, Cubey et al. 2014; Shearer and Ranney 2013, Wikipedia 2014). Those names are currently invalid since, according to the ICN, all proposed scientific names, including hybrid names, require that they be formally published and described and be represented by a type specimen. A type specimen is the specimen to which the name is permanently attached and which is publicly available for consultation (McNeill et al. 2012).

The name Cornus × rutgersensis is proposed for the hybrid *Cornus kousa* × *Cornus florida*. A new name is also proposed, Cornus × elwinortonii, honoring our colleague Dr. Elwin Orton, for the hybrid he created between *Cornus kousa* and *Cornus nuttallii*. Full morphological descriptions, typification, illustrations, horticultural information with cultivar names, disease response, and a discussion on the formation of intermediate morphological traits with regard to leaf size, inflorescence structure, and fruits are provided for each of these new names. In doing this, we provide both formal names and summarize information of general botanical interest of these popular garden plants for botanists and horticulturalists.

## Taxonomic treatment

### Cornus × elwinortonii

Taxon classificationPlantaeORDOFAMILIA

Mattera, T. Molnar, & Struwe hybr. nov.

urn:lsid:ipni.org:names:77148930-1

**Figure 1.**
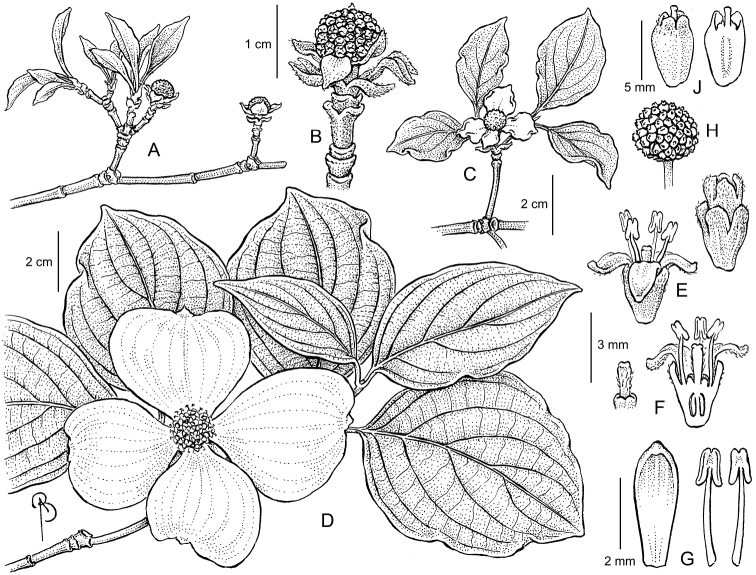
Illustration of Cornus × elwinortonii *‘KN4-43’, PP 16293, Starlight*®. **A** Branch, showing expanding leaf and floral bract tissues in the spring **B** Close up of inflorescent bud prior to complete bract and leaf expansion **C** Node, showing fully expanded leaves and partially expanded floral bracts. **D** Branch, showing inflorescence with flowers in full bloom; floral bracts fully expanded **E** Close up of flower at dehiscence, note synsepalous calyx and apopetalous corolla **F** Dissected flower, showing single gynoecium **G** Close up of petal and stamens, note dehiscence occurs longitudinally **H** Single inflorescence, showing many tightly compressed parthenocarpic drupes **J** Single drupe, showing compressed form and protruding style. Drawings by Bobbi Angell from the holotype.

**Figure 2.**
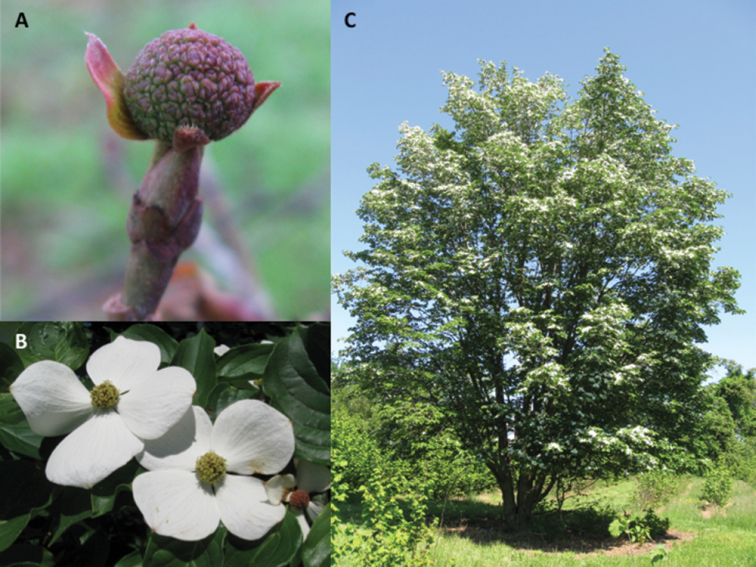
Photos of Cornus × elwinortonii. **A** Close up of dormant inflorescent bud; note the exposed flower buds and partially developed floral bracts **B** Flowers and floral bract display after dehiscence; note frost damaged inflorescence on the far right **C** Habit of mature plant. Photograph by Thomas Molnar.

 Orton’s dogwood Figs 1 , 2

#### Diagnosis.

Cornus × elwinortonii is similar to both *Cornus kousa* and *Cornus nuttallii* but differs in its intermediate flower number per inflorescence and in its intermediate tree height. Cornus × elwinortonii has 55-80 flowers per head, whereas *Cornus kousa* has 20–60, and *Cornus nuttallii* has 70–100. Cornus × elwinortonii is also intermediate in plant height, with a maximum of 10 m height (*Cornus kousa* reaches 6 m height, while *Cornus nuttallii* is 12–23 m tall as a mature tree).

#### Type.

USA. New Jersey: New Brunswick, Middlesex County, Ryders Lane, Horticultural Farm 1, original tree (ramet) of ‘KN4-43’ Starlight®, cultivated plant in open field adjacent to Rutgers Equine research farm, surrounded by hazelnut (*Corylus* spp.) trees planted in rows, GPS location (WGS84) 40.4676N, -74.4281E, 18 m, 17 May 2014, *R. Mattera 33* (holotype: NY, isotypes: CHR, JEPS, MO, US, to be distributed).

#### Description.

Tree with upright or rounded habit,10 m in height at maturity. Bark rough, as sandpaper, with exfoliation at the base of the trunk; lenticels abundant, 1.25–1.75 × 0.40–0.65 mm. Leaves opposite, simple, elliptic, ovate to obovate, 10.3–15.3 × 5.9–9.1 cm; base attenuate to oblique; margin entire to slightly wavy, cuneate/crenate; apex apiculate; venation with 5 (or 6) pairs of secondary veins; midrib and abaxial surfaces with conspicuous indumentum of short, fine, downy, whitish beige trichomes with occasional dark tufts of longer brown trichomes in the axils of midvein and secondary veins, indumentum less dense on adaxial surfaces. Overwintering inflorescence buds not covered by the two outer opposing pairs of vegetative bracts, minimally covered by two inner opposing pairs of floral bracts (0–40% coverage; floral bracts more developed than in *Cornus kousa* during overwintering). Inflorescence capitate, globose, with 55–80 sessile flowers per head, subtended by 4 (rarely 5 or 6) simple entire, decussate pairs of bracts. Bracts petaloid at anthesis, ovate to lanceolate, sometimes wider than long, overlapping or not when fully developed, 5–8 cm long, 3.5–7.0 cm wide, usually white, or occasionally pink; base tapering to point of attachment; apex acuminate to cuspidate. Peduncle 1.5–8.0 cm long at time of flowering. Flowers actinomorphic, bisexual, 4-merous. Calyx lobes ovate; apex obtuse. Corolla lobes obovate, apex slightly acute. Stamens 4, exserted from corolla mouth, inserted in corolla lobe sinuses; filaments 1.5–2.5 mm long, 0.2–0.5 mm wide; anthers ovoid, bae sagittate, longitudinally dehiscent, 1.0–1.1 × ca. 0.25 mm; pollen less prevalent on hybrids compared to parent species, white or yellow-brown. Gynoecium epigynous, with nectar disc; ovary syncarpous; style 1, 1.5–2.5 mm long, exserted from corolla; stigma indistinct, ca. 0.4 mm long. Fruit either many drupes tightly compressed together, or a multiple fruit formed from 1-seeded drupelets forming a mounded raspberry-like fruit, often parthenocarpic.

#### Parent source material.

The parents of the F_1_ hybrid (‘KN4-43’ Starlight®) are *Cornus kousa* ‘Simpson No. 1’ (female), an unpatented cultivar received from Tennessee Valley Nursery (Winchester, TN, USA) and planted at Rutgers Gardens (New Brunswick, NJ) on 16 April 1970, and *Cornus nuttallii* ‘Goldspot’ (male), received from Alfred Teufel Nursery (Portland, OR, USA) and planted in 1972.

#### Ecology and phenology.

In New Jersey, Cornus × elwinortonii flowers during May and June, and the fruit matures from September to October. Various beetles and bees visit the flowers at anthesis, with an abundance of goldenrod soldier beetles (*Chauliognathus pensylvanicus*) frequently observed by the authors. The mostly sterile fruit with little pulp generally senesces and falls from the trees by October. The few fruits with a developing seed are swollen and have more pulp. We suspect they are eaten by insects and birds.

#### Etymology.

The epithet, *elwinortonii*, honors the prominent dogwood breeder Dr. Elwin Orton (b. 1930), Professor Emeritus in the Department of Plant Biology and Pathology at Rutgers University. He was the first to successfully develop and release a hybrid between *Cornus kousa* and *Cornus nuttallii*. The common name, Orton’s dogwood, is proposed for this hybrid.

#### Distribution.

Cornus × elwinortonii is known only from cultivation, although at times it produces viable seeds. The natural range of the staminate parent, *Cornus nuttallii*, is in western North America from the lowlands of British Columbia (Canada) to southern California (USA), with a small isolated population in northern Idaho (USA, Keir et al. 2011, Klinka et al. 2000). The other parent, *Cornus kousa*, is native to mesic forests of Japan, Korea and China (Flint 1997, Xiang and Boufford 2005). *Cornus nuttallii* cannot withstand sustained periods of frost, thus limiting its natural and cultivated range. In contrast, *Cornus kousa* can be cultivated throughout much of the USA; Europe, and Asia in U.S. Department of Agriculture cold hardiness zones 6a-9a (Daly et al. 2012, Flint 1997). The hybrid Cornus × elwinortonii can survive sustained frosts and has a similar climate range as *Cornus kousa*. However, for some cultivars of Cornus × elwinortonii the floral buds are less cold hardy than in the parent *Cornus kousa*. In colder climates, including in New Brunswick, NJ, where the hybrid originated, flower buds can be damaged by cold winter temperatures, leading to a reduced floral bract display in the spring (E. Orton personal communication).

#### Horticulture.

Plants of Cornus × elwinortonii are grown as landscape ornamentals and can be cultivated wherever *Cornus kousa*, *Cornus nuttallii*, and *Cornus florida* may be grown. This hybrid is cultivated for its all-year round appeal: floral bracts, attractive foliage, autumn color and appealing bark (Eberts 2007) Cornus × elwinortonii is typically propagated asexually through budding and grafting on seedling rootstocks of *Cornus kousa* or *Cornus florida*. Patented and trademarked cultivars that belong to this hybrid include ‘KN4-43’ Starlight® (F_1_), ‘KN 30-8’ Venus® (first backcross to *Cornus kousa*), and ‘KN144-2’ Rosy Teacups® (third serial backcross to *Cornus kousa*; Table 1). We know of no other commercially available cultivars of Cornus × elwinortonii.

#### Disease response.

While dogwood anthracnose caused by the fungus *Discula destructiva* Redlin is known to infect and kill *Cornus nuttallii*, it has not been reported to be a significant problem on either *Cornus kousa* or Cornus × elwinortonii (Daughtrey and Hibben 1994; Fulcher et al. 2012; Hagan et al. 1998).

#### Additional material provided.

Additional collections from the same individual as the holotype specimen, but on different dates (*R. Mattera 27*, *R. Mattera 29*, *R. Mattera 31*, and *R. Mattera 35*) will all be deposited at CHR, NY, and MO).

### Cornus × rutgersensis

Taxon classificationPlantaeORDOFAMILIA

Mattera, T. Molnar & Struwe hybr. nov.

urn:lsid:ipni.org:names:77148931-1

**Figure 3.**
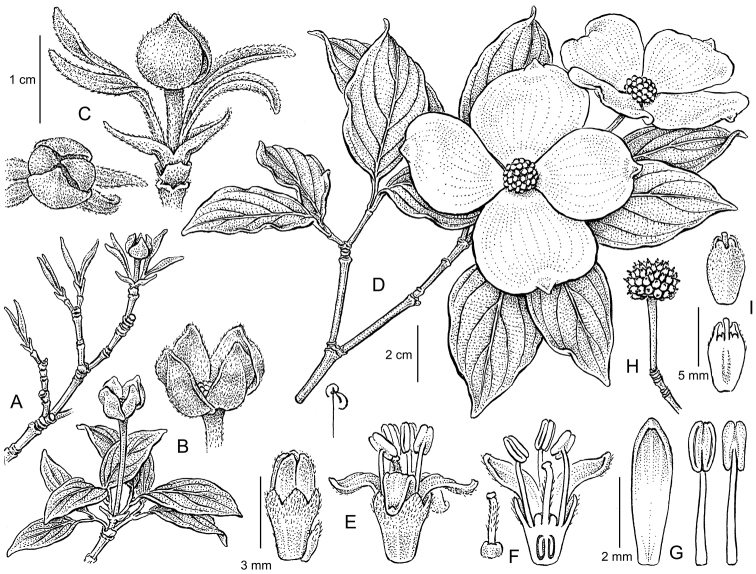
Illustration of Cornus × rutgersensis *Cornus ‘Rutgan’, PP7207, Stellar Pink®.* **A–B** Branch, showing expanding leaf and opening of floral bract tissues in the spring **B** Close up of inflorescent bud prior to complete bract and leaf expansion **C** Close up of single inflorescence post bud-break, showing pair of unexpanded floral bracts clinging to flower head; note pair vegetative bracts still attached at base of inflorescence **D** Branch, showing inflorescence with flower buds still closed; floral bracts fully expanded **E** Close up of flower, showing both before and after anthesis; note synsepalous calyx, apopetalous corolla and exerted stamens **F** Dissected flower, showing single gynoecium and exerted style **G** Close up of petal and stamens, note dehiscence occurs longitudinally **H** Single inflorescence, showing many tightly compressed parthenocarpic drupes **I** Single drupe, showing compressed form and protruding style. Drawings by Bobbi Angell from the holotype.

**Figure 4.**
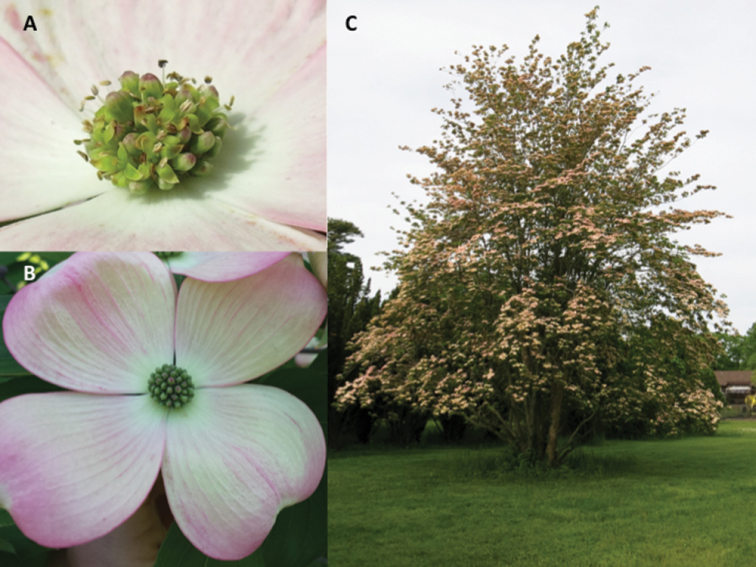
Photos of Cornus × rutgersensis. **A** Close up of inflorescence, showing varying stages of flowering **B** Inflorescence with full floral bract display and flowers before anthesis **C** Habit of mature plant. Photographs **A** and **C** by Thomas Molnar; photo **B** by Robert Mattera.

**Figure 5.**
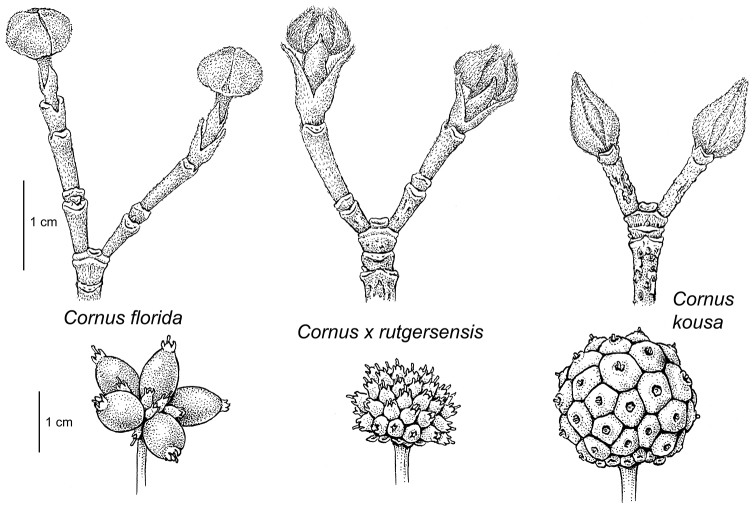
Comparison of flowering bud and fruit development in *Cornus florida*, Cornus × rutgersensis, and *Cornus kousa*. Drawing by Bobbi Angell.

 Rutgers’ dogwood Figs 3 , 4 , 5

#### Diagnosis.

Cornus × rutgersensis is similar to *Cornus kousa* and *Cornus florida*, but differs in its intermediate leaf size and fruit aggregation and size. Cornus × rutgersensis has leaves 9.0–16.8 × 4.2–9.1 cm, whereas the leaves of *Cornus kousa* are 5.1–10.2 × 2–5 cm and for *Cornus florida* 7.6–15.2 × 2–7 cm). Cornus × rutgersensis forms many single-seeded parthenocarpic drupes 0.5 × 0.25 mm wide, but does not form a multiple fruit as in *Cornus kousa*. *Cornus florida* has larger, fertile drupes 13–18 × 6–9 mm.

#### Type.

USA: New Jersey: New Brunswick, Middlesex County, Ryders Lane, Rutgers Gardens, original tree (ramet) of ‘Rutgan’ Stellar Pink®, cultivated plant in open grass field behind Rutgers Ornamental Horticultural Field lab, adjacent to a pine tree windscreen, GPS (WSG84) 40.4732N, -74.4238E, 22 m, 25 May 2014, *R. Mattera 34*, holotype (NY), isotypes (CHR, JEPS, MO, US, to be distributed).

#### Description.

Trees with upright or rounded habit, F_1_ hybrids cultivated at Rutgers range from 3 –10 m in height at maturity. Bark smooth when young, light gray to brown older bark exfoliating; lenticels on young bark abundant, 0.5–0.7 × 0.3–0.4 mm. Leaves opposite, simple, ovate to elliptic, 9.0–16.8 × 4.2–9.1cm; base attenuate, cuneate-crenate to oblique; margin entire to moderately wavy; apex apiculate or acuminate; with 5 pairs of secondary veins; abaxial surface smooth; indumentum of many white trichomes on both surfaces, abaxial margin with many white trichomes, with dark tufts of trichomes along midrib and veins. Overwintering inflorescence buds intermediate in size and developmental structure between the parents. Outermost vegetative bracts barely covering the inflorescence; inner two pairs of floral bracts enclosing flower head; unlike in either parent, floral bracts covering only 10–45% of the flower head. Inflorescence capitate, globose, with 30–50 flowers per head, surrounded by 4 floral bracts; floral bracts sessile, entire, in decussate pairs, petaloid at anthesis, ovate to lanceolate, sometimes wider than long, overlapping or not; 4.0–6.5× 3–6 cm, white or pink; base tapering to point of attachment, apex acuminate to cuspidate. Peduncle 3.5–7.5 cm long at time of flowering. Flowers actinomorphic, bisexual; 4-merous. Calyx lobes ovate, acute. Corolla lobes obovate, slightly acute. Stamens 4, exserted, inserted in corolla lobe sinuses; filaments 2.7–4.5 mm long, 0.2–0.3 mm wide; anthers longitudinally dehiscent, 0.4–2.0 × 0.5-0.8 mm; pollen yellowish brown. Gynoecium epigynous, with nectar disc; ovary syncarpous; style 1, inserted to exserted from corolla mouth, 1.5–1.9 × 0.3–0.5 mm; stigma slightly capitate, ca. 0.25 mm long. Fruit single drupes, rarely fused into a multiple fruit; fruits often formed without proper seed development (i.e., sterile fruits), if fertile, then 1-seeded.

#### Parent source materials.

The parents of the described type F_1_ hybrid (‘Rutgan’ Stellar Pink®) are *Cornus kousa* K2 (female) grown at Rutgers Gardens from a seedling received from Ben C. Blackburn, Willowwood Arboretum (Gladstone, NJ) in May of 1949, and *Cornus florida* ‘Sweetwater Red’ (male), received from Boyd Nursery (McMinnville, TN) and planted at Rutgers Gardens.

#### Ecology and phenology.

Cornus × rutgersensis flowers in New Jersey (USA) in May; the fruits mature from September to October. Adrenid and halictid bees and cerambycid beetles pollinate the flowers of *Cornus kousa* while only adrenid and halictid bees pollinate *Cornus florida* (Rhoades et al. 2011). It is believed that the same insects visit the flowers of the hybrid. All cultivars released to the public, except ‘KF111-1’ Hyperion® (first backcross to *Cornus kousa*), are sterile. Sterile specimens produce very little pulp in the fruit and no fully formed seeds. It is unlikely that these aborted fruits serve as a significant food source for insects or birds. Hyperion® produces fruits that are more similar to *Cornus kousa* and likely serve as a food source for wild animals, although there are no studies to substantiate this assumption.

#### Etymology.

The epithet *rutgersensis* is based on Rutgers University, The State University of New Jersey, the academic home of Dr. Elwin Orton’s dogwood breeding program, which is now continued by co-author Thomas Molnar. Rutgers University was founded in 1766 in New Brunswick, NJ, and was named in 1825 after Colonel Henry Rutgers, a US Revolutionary War veteran (Rutgers University 2014). We suggest the common name Rutgers’ dogwood for this hybrid.

#### Distribution.

Cornus × rutgersensis is known only from cultivation. One of the parent species, *Cornus florida*, an understory tree in mesic forests (Fulcher et al. 2012, Hillier Nurseries 2002, Porter 1903, Schwartz 1994, Wennerberg 2006), ranges from southern Maine to Florida, and as far west as Texas in the USA (Mohlenrock 2006, Schwartz 1994, Wennerberg 2006). *Cornus kousa* occurs in mesic forests in Japan, Korea, and China (Flint 1997, Xiang and Boufford 2005). No formal studies have been done to determine climate range for Cornus × rutgersensis; however, it is generally believed that its range is similar and intermediate between the two parent species *Cornus florida* and *Cornus kousa*.

#### Horticulture.

Cornus × rutgersensis is grown as a landscape ornamental and, in general, can be cultivated wherever *Cornus florida* or *Cornus kousa* can be grown. Cornus × rutgersensis is typically propagated asexually through budding and grafting on seedling rootstocks of *Cornus kousa* or *Cornus florida*. The cultivars ‘KF1-1’ Saturn®, ‘Rutban’ Aurora®, ‘Rutcan’ Constellation®, ‘Rutdan’ Celestial,®, ‘Rutfan’ Stardust®, ‘Rutgan’ Stellar Pink®, and ‘Rutlan’ Ruth Ellen® are all direct F_1_ hybrids of *Cornus florida* and *Cornus kousa*, and all produce sterile fruit. ‘KF111-1’ Hyperion® is a first backcross to *Cornus kousa* and produces some fertile fruit. We know of no other commercially available plants of Cornus × rutgersensis.

#### Disease response.

Cornus × rutgersensis shows resistance to dogwood anthracnose and resistance or high levels of tolerance to powdery mildew (*Erysiphe pulchra* and *Phyllactinia guttata*; Li et al. 2009, Ranney et al. 1995, Trigiano et al. 2005).

#### Additional material examined.

Additional collections from the same individual from which the holotype was collected, but at other dates: *R. Mattera 26*, *R. Mattera 28*, *R. Mattera 30*, *R. Mattera 32*, will all be deposited at CHR, NY, and MO).

## Discussion

**Morphological intermediacy in hybrids.** Interspecific hybrids are commonly intermediate in their morphology between their parents (e.g., Tovar-Sanchez and Oyama 2004). However, in hybrids between the big-bracted dogwoods, there is the added complication of the parental species having either a multiple, berry-like fruit or single-seeded drupes, and remarkably different inflorescence buds, bract morphology and phenological development. Despite such large differences, the hybrids clearly express intermediate phenotypes and provide good examples of ‘halfway’ morphologies created through hybridization. Intermediate traits include leaf size, inflorescence structure, and fruit type, which are three important ornamental characteristics of big-bracted dogwoods. The shape of the bract shape is also intermediate in these hybrids, leading to increased variation in bract shape. Also, the intermediate flowering times allow for a lengthening of the display of the ornamental bracts across the big-bracted clade. Both hybrids discussed in this paper also display novel characteristics not seen in previous dogwood cultivars. For example, ‘KN30-4’ Venus® displays larger floral bracts than in other hybrid cultivars or in the species of *Cornus* known to us.

Generally, Cornus × rutgersensis and its parents display similar tree shape and form, but the hybrid displays increased vigor and growth (Fig. 4). In *Cornus × elwinortonii*, tree shape and growth habit appear similar to the parents, *Cornus kousa* and *Cornus nuttallii*, but the hybrid is significantly more vigorous than *Cornus kousa* and shows increased growth in younger trees. Cornus × elwinortonii can be significantly larger in stature (to 8 m; Fig. 2) than most trees of *Cornus kousa* (to 6 m; Gilman and Watson 1993b), but hybrid tends to be significantly shorter than *Cornus nuttallii* (to 12 m, occasionally to 22.9 m; Gucker 2005).

The leaves of Cornus × rutgersensis are intermediate between the two parents, being longer and wider than *Cornus kousa* and shorter and narrower than *Cornus florida*. A similar phenomenon was recorded in the *Quercus crassifolia* Bonpl. × *Quercus crassipes* Bonpl. hybrid complex (Fagaceae; Tovar-Sanchez and Oyama 2004) and in crosses between the herbs *Brassica oleracea* L. and *Sinapis alba* L. (Brassicaceae; Hansen and Earle 1996). The leaves of *Cornus nuttallii* and *Cornus kousa* are narrower (5-7 cm) than their offspring, *Cornus × elwinortonii* (5-8 cm), and the leaves of the hybrid can also have a crinkled appearance, which is not characteristic of either parent. Such novel hybrid characteristics are not unusual and have also been reported in the *Quercus crassifolia* × *Quercus crassipes* complex (Tovar-Sanchez and Oyama 2004) and in *Carica papaya* L. × *Vasconcellea cauliflora* (Jacq.) A.DC. (reported as *Carica cauliflora* Jacq.; Caricaceae; Magdalita et al. 1996).

Inflorescence bud morphology and development shows dramatic differences between the parents of Cornus × rutgersensis and is also correlated with large differences in floral bract display (and anthesis). The floral bracts of *Cornus florida* are displayed before vegetative bud-break in early spring, whereas in *Cornus kousa* the floral bracts are displayed after the foliage is fully developed. In *Cornus florida*, the inflorescence bud consists of two pairs of floral bracts (inner and outer) tightly clinging to a well-developed inflorescence head. Underdeveloped vegetative bracts are present but do not cover the inflorescence. *Cornus kousa* has two pairs of floral bracts that tightly cling to the underdeveloped inflorescence. In addition, they are tightly covered by two pairs of vegetative bracts. The hybrid displays an intermediate flower bud in which floral bracts cling to the inflorescence and vegetative bracts cling loosely to the flower head (Fig. 3c and 5). As expected, intermediate inflorescence bud development leads to intermediate floral bract display and flowering time. The flowering period of Cornus × rutgersensis ranges from the end of flowering in *Cornus florida* to the beginning of flowering in *Cornus kousa*. Correlation of morphological variation in floral bud shape to intermediate flowering time has also been reported in hybrids between *Fraxinus excelsior* L. and *Fraxinus angustifolia* Vahl (Oleaceae; Gerard et al. 2006). Notably, in Cornus × rutgersensis, the floral bracts tend to only weakly cover the inflorescence during overwintering, resulting in 10–45% of the flowers being naked (exposed).

Differences in the inflorescence buds also exist for Cornus × elwinortonii and its parents, *Cornus nuttallii* and *Cornus kousa*. The underdeveloped inflorescence head in *Cornus kousa* is tightly covered by two pairs of floral and vegetative bracts. *Cornus nuttallii* has a completely exposed inflorescence head, where the small floral and vegetative bracts do not cover the developing flower buds. *Cornus nuttallii*, native to the Pacific Northwest (USA), is exposed to milder winter temperatures than *Cornus kousa* from eastern Asia. Cornus × elwinortonii displays an intermediate bud ranging from completely exposed to completely covered. In Cornus × rutgersensis, there is strong variation in the degree of coverage by the bracts, with 10-45% naked to nearly completely covered floral buds.

Distinct differences between inflorescence architectures can also be observed between parents and their hybrids. In *Cornus kousa*, all flowers in the inflorescence are fused, creating a densely merged ball of flowers, while in *Cornus florida* the flowers are not fused, creating a more open structure. The flowers in their hybrid, Cornus × rutgersensis, are densely packed and at first appear to be fused together; however, they are separate even if closely positioned (Fig. 3 and Fig. 4A). The number of flowers in each inflorescence varies greatly within big-bracted dogwoods, from a few dozen to over one hundred. The hybrids also show intermediacy in the number of flowers: Cornus × rutgersensis (30–50 flowers/head) from parents *Cornus florida* (20–30) and *Cornus kousa* (20–50), and Cornus × elwinortonii (55–80) from parents *Cornus kousa* (20–60) and *Cornus nuttallii* (70–100).

In *Cornus florida*, the individual flowers develop into single-seeded drupes, while in *Cornus kousa* the fused flowers develop into single-seeded druplets that are fused into a multiple, berry-like fruit (Fig. 5). The nearly always sterile hybrid Cornus × rutgersensis may produce parthenocarpic fruit displaying intermediate characteristics (Fig. 5). Fruits containing seeds swell and develop into individual drupes or drupelets. This is the only example we know of where a hybrid has been created between parents with single and multiple fruit types. The hybrid between *Jatropha curcas* L. and *Jatropha integerrima* Jacq., formed from the crossing of plants with large drupaceous fruits (*Jatropha curcas*) and small deeply lobed capsules, displayed an intermediate fruit shape between two different fruit types as well (Rupert et al. 1970; Sujatha and Prabakaran 2002).

**Success of hybrids.** Ornamental plants play an important role in society, providing aesthetic value, shade, wildlife habitat and food, and soil stabilization. As popular ornamental trees in temperate and sub-tropical regions worldwide, improved cultivars of big-bracted dogwoods are desired. Demand for novel, vigorous, and disease-resistant plant material is high; however, limited genetic variability can exist for some traits. For example, there are only a few cultivars of *Cornus florida* that express resistance to powdery mildew (Windham et al. 2003, Windham and Witte 1998) and the floral bracts of *Cornus kousa* and *Cornus nuttallii* lack the dark red of the most successful *Cornus florida* cultivars (Cappiello and Shadow 2005, Dirr 2009). Orton’s use of interspecific hybridization to develop the novel plants described here (Cornus × rutgersensis and Cornus × elwinortonii) resulted in the successful development of cultivars with enhanced aesthetic qualities and improved disease resistance.

Upon its introduction to the US from Asia, dogwood anthracnose devastated natural stands of *Cornus florida*, a plant species highly susceptible to this fungal disease. For example, mortality rates as high as 86% occurred in a ten year period in Connecticut (Holzmueller et al. 2006). *Cornus nuttallii* is also highly susceptible to dogwood anthracnose. The Asian dogwood *Cornus kousa* occurs sympatrically with the causal agent of dogwood anthracnose *Discula destructiva* in Asia, and most cultivars of *Cornus kousa* have a high level of tolerance or resistance to this disease (Hibben 1990, Ranney et al. 1995). Because of results from field evaluations and the *Cornus kousa* parentage, all of Orton’s hybrids were believed to be highly resistant to this disease at the time of their commercial release. The Stellar® Series and Jersey Star® releases came at a time when disease incidence was high in the United States. However, Ranney et al. (1995) showed that not all of the Rutgers hybrids maintained resistance over the years, although some still displayed tolerance.

Powdery mildew, believed to be introduced from Asia, is less devastating to natural stands of *Cornus florida*. Instead, this disease has strongly impacted the nursery industry, raising production costs and reducing aesthetic appeal. Cultivars of *Cornus florida* display little resistance to this fungal disease. Of more than 100 available cultivars of *Cornus florida* (Santamour and McArdle 1985), only five (‘Jean’s Appalachian Snow’, ‘Karen’s Appalachian Blush’, ‘Kay’s Appalachian Mist’, ‘Appalachian Joy’ and ‘Cherokee Brave’) display high levels of tolerance or resistance to powdery mildew (Li et al. 2009, Ranney et al. 1995). Again, cultivars of *Cornus kousa* generally show high levels of tolerance (Li et al. 2009, Ranney et al. 1995). Due to Orton’s selection of parents, several cultivars of Cornus × rutgersensis (e.g., Stellar Pink®, Aurora®, Stardust®, Celestial®, and Constellation®) are resistant to powdery mildew (Li et al. 2009).

**Scientific naming of horticultural plants.** Crucial to communication in all parts of our lives is the naming of objects and phenomena. We need words to tell other people what we are talking about, and the words need to have uniform and clear meanings. For botany, our scientific names form such a uniform language that is universal and used in fields including biodiversity inventories, phytochemistry, horticulture, crop plants, and other scientific and/or economic endeavors. Many scientific plant names are listed in the International Plant Names Index (http://www.ipni.org) and in other resources such as floras, dictionaries, The Plant List (http://www.theplantlist.org/), RHS Plant Finder (http://www.rhs.org.uk/plants/), Encyclopedia for Life (http://eol.org), Wikipedia (http://wikipedia.com). Unfortunately, many misspelled, outdated, unpublished, illegitimate, and invalid names are still in use worldwide in popular literature, websites, and non-taxonomic publications, especially for commonly cultivated and medicinal plants (Bennett and Balick 2014 for examples, see Struwe 2014).

It can be argued that we do not need formal scientific names for all artificially created hybrid plants, since cultivar and trademark names exist and names of cultivated plants follow *The International Code of Nomenclature for Cultivated Plants* (ICNCP, Brickell et al. 2009). However, names of hybrids following the *International Code for algae, fungi, and plants* may be useful when cataloging species diversity, natural or human-made, and linking hybrids with their parental species. Cornus × rutgersensis is a name already in use on a global scale, but was never proposed formally according to the rules of the ICN. Validating this name is the simplest way to provide an acceptable and useful name to the horticultural community. Since the second hybrid, Cornus × elwinortonii, is also a commonly grown and well-known hybrid in gardens, to propose it formally is also useful. Even if self-propagating seedlings from these hybrids are not known, we do know that viable seeds are sometimes produced; making it is possible that spontaneous progeny will arise in the future

## Summary

The hybrids Cornus × rutgersensis (*Cornus florida* × *Cornus kousa*) and Cornus × elwinortonii (*Cornus kousa* × *Cornus nuttallii*) were developed at Rutgers University by Dr. Elwin Orton, and are good examples of controlled hybrid crosses showcasing intermediate morphological and phenological characteristics for leaf size, inflorescence bud structure, flowering time, and fruit structure. The horticultural success of big-bracted dogwood hybrids in the nursery and landscape industry can largely be attributed to their inherent disease resistance and enhanced aesthetic qualities that represent novel intermediate phenotypes between their parent species.

## Supplementary Material

XML Treatment for Cornus × elwinortonii

XML Treatment for Cornus × rutgersensis
